# MAGEB2-Mediated Degradation of EGR1 Regulates the Proliferation and Apoptosis of Human Spermatogonial Stem Cell Lines

**DOI:** 10.1155/2023/3610466

**Published:** 2023-06-02

**Authors:** Xueheng Zhao, Zenghui Huang, Yongzhe Chen, Qianyin Zhou, Fang Zhu, Huan Zhang, Dai Zhou

**Affiliations:** ^1^Institute of Reproduction and Stem Cell Engineering, School of Basic Medicine Science, Central South University, Changsha, Hunan 410000, China; ^2^Reproductive & Genetic Hospital of CITIC-Xiangya, Changsha, Hunan 410000, China; ^3^First Affiliated Hospital of University of South China, Hengyang, Hunan 421000, China; ^4^College of Life Sciences, Hunan Normal University, Changsha, Hunan 410000, China; ^5^Clinical Research Center for Reproduction and Genetics in Hunan Province, Changsha, Hunan 410000, China

## Abstract

Spermatogonial stem cells are committed to initiating and maintaining male spermatogenesis, which is the foundation of male fertility. Understanding the mechanisms underlying SSC fate decisions is critical for controlling spermatogenesis and male fertility. However, the key molecules and mechanisms responsible for regulating human SSC development are not clearly understood. Here, we analyzed normal human testis single-cell sequencing data from the GEO dataset (GSE149512 and GSE112013). Melanoma antigen gene B2 (MAGEB2) was found to be predominantly expressed in human SSCs and further validated by immunohistology. Overexpression of MAGEB2 in SSC lines severely weakened cell proliferation and promoted apoptosis. Further, using protein interaction prediction, molecular docking, and immunoprecipitation, we found that MAGEB2 interacted with early growth response protein 1 (EGR1) in SSC lines. Reexpression of EGR1 in MAGEB2 overexpression cells partially rescued decreased cell proliferation. Furthermore, MAGEB2 was shown to be downregulated in specific NOA patients, implying that abnormal expression of MAGEB2 may impair spermatogenesis and male fertility. Our results offer new insights into the functional and regulatory mechanisms in MAGEB2-mediated human SSC line proliferation and apoptosis.

## 1. Introduction

Infertility is a global health problem that hinders about 15% of couples trying to conceive, and about 50% of these cases are caused by male factors [1]. Nonobstructive azoospermia (NOA) is among the most severe and difficult-to-treat forms of male infertility. NOA is usually categorized into pretesticular and testicular based on the location of the etiology. Pretesticular NOA arises because of hormonal abnormalities. Testicular NOA is associated with intrinsic testicular defects that lead to impaired spermatogenesis [2]. Based on histological features, NOA can be classified as maturation arrest, hypospermatogenesis, and Sertoli cell only. Additionally, mixed histological patterns are common in men with NOA. The etiology of NOA is highly intricate, which seriously affects the treatment [3]. Although few NOA patients can obtain sperm through microsurgical testicular sperm extraction and have offspring using assisted reproductive techniques, most of them cannot obtain genetic offspring [2]. How to address the fertility needs of NOA patients is a challenge in clinical practice. Stem cells can self-renew and restore the function of tissues and organs. In mice, the reproductive lineage can be reconstituted by transplantation of spermatogonial stem cells (SSCs) [4]. However, this clinically meaningful experiment is currently not accessible to humans due to the weak proliferation potential of human SSCs in vitro [5].

Numerous vital molecules and signaling pathways in mice that regulate SSC self-renewal and differentiation have been identified [6, 7]. GDNF has been shown to be a key regulator in maintaining and promoting SSC self-renewal [8], and a cell culture system with the addition of GDNF, fibroblast growth factor 2 (FGF2), and epidermal growth factor (EGF) can maintain long-term expansion in vitro of mouse SSCs [9]. The fate decisions of SSC have also been reported to be controlled by niche and intrinsic factors [7]. Niche factors, including GDNF [10–12], FGF2 [13], and C-X-C motif chemokine ligand 12 (CXCL12) [14], are generally thought to promote self-renewal and proliferation of SSC in mice. WNT signals are usually considered to promote the proliferation of undifferentiated spermatogonia [15, 16]. Still, it was also reported to promote the differentiation of SSCs to progenitor cells [17]. On the other hand, retinoic acid (RA) is commonly thought to contribute to SSC differentiation [18, 19]. Many intrinsic factors, including promyelocytic leukemia zinc finger (PLZF) [20, 21], forkhead box protein O1 (FOXO1) [22], Nanos homolog 2 (NANOS2) [23, 24], and inhibitor of DNA binding 4 (ID4) [25, 26], are involved in SSC self-renewal and proliferation. There are also many molecules that have been found to promote SSC differentiation, including neural precursor cells express developmentally downregulated protein 4 (NEDD4) [27], sal-like protein 4 (SALL4) [28], and stimulated by retinoic acid gene 8 (STRA8) [29, 30]. In addition, many epigenetic aspects are involved in the regulation of SSCs, which are not elaborated here.

Based on studies in mice, attempts have been made to culture human SSCs in vitro, and our previous study reported the roles of ASB9 [31], SPOCD1 [32], and TCF3 [33] in the human SSCs. However, many regulatory molecules and signaling pathways are not conserved due to species differences between humans and mice. For example, octamer-binding protein 4 (OCT4) was reported to be involved in the self-renewal of mouse SSCs, but it is not present in human SSCs [34, 35]. FBXW7 has been reported to be predominantly expressed in mouse SSCs and negatively regulates its self-renewal [36]. However, our previous findings indicated that FBXW7 was widely expressed in human spermatogonia and spermatocytes [37]. In addition, the classification of human spermatogenic epithelium [38] and SSCs [39] also differed significantly from that of mice.

Taking into account the species differences between humans and rodents, the use of human samples to study the mechanisms underlying SSC fate decisions is certainly the best option. Recently, the advent of single-cell RNA sequencing (scRNA-seq) has facilitated our study of human SSCs [40–43], allowing us to understand the transcriptional profile of SSCs at the level of individual cells and identify rare cells in the transition state further and explore the regulatory mechanisms of SSC by studying changes in transcription. To overcome the shortcomings of the animal model results, we analyzed data from adult testis single-cell sequencing of GSE149512 [41] and GSE112013 [40] and then extracted transcriptional data of human SSCs. Further analysis identified many genes and signaling pathways associated with human SSC self-renewal and proliferation, including melanoma antigen gene B2 (MAGEB2), SPOCD1, CST3, and ID4.

In this study, our findings suggested that MAGEB2 is predominantly localized in human SSC using scRNA-seq analysis and immunohistochemistry. Overexpression of MAGEB2 in human SSC lines significantly impaired cell proliferation and induced apoptosis. The molecular docking and immunoprecipitation results indicated a protein interaction between MAGEB2 and EGR1. MAGEB2-mediated reduction of cell proliferation can be partially rescued by the reexpression of EGR1 in human SSC lines. Furthermore, we found downregulation of MAGEB2 protein in the testes of some NOA patients. Our data revealed the roles and mechanisms of MAGEB2-mediated human SSC line proliferation and apoptosis, which provided novel insights into the regulatory mechanisms of human SSC lines.

## 2. Materials and Methods

### 2.1. Sources and Analysis of Single-Cell RNA Sequencing Data

The normal testicular cell scRNA-seq datasets GSE112013 (three samples) [40] and GSE149512 (five samples) [41] were downloaded from the GEO dataset (https://www.ncbi.nlm.nih.gov/gds). To analyze the scRNA-seq data, Seurat 4.3.0 (http://satijalab.org/seurat/) was used. In brief, we first read the expression matrix data through the Read10X and read.table function and create a Seurat object for each expression matrix. Then, the Seurat object data were filtered and normalized, and cells with gene count less than 500 and a percentage of mitochondrial genes higher than 20% were removed, and ribosomal genes were manually eliminated. After this, the IntegrateData function was used to merge all data and remove batch effects after finding variable features of each object. Next, the integrated Seurat object was subjected to downscaling and clustering analysis using uniform manifold approximation and projection (UMAP), and each cluster was identified by marker genes. Furthermore, developmental trajectory analysis of SSCs was performed using Monocle 3 (https://cole-trapnell-lab.github.io/monocle3/). All the point and line plots were optimized using ggplot2 (https://www.rdocumentation.org/packages/ggplot2, version 3.4.0).

### 2.2. Collection of Human Testes Tissue

Our study was approved and supervised by the Ethics Committee of Reproductive & Genetic Hospital of CITIC-Xiangya (LL-SC-2021-025); all participants participated in the study by signing an informed consent statement. Human testicular samples were obtained from 18 NOA patients undergoing mTESE surgery, aged 28 to 48. The samples were collected after being used for treatment, with approximately 25 mg remaining and anonymized.

### 2.3. Source and Culture of Human SSC Lines

The human SSC lines was established by Hou et al. [44], and it was a gift from Prof. He's lab (Hunan Normal University, Changsha, China). In brief, the SSC lines were established by overexpressing human SV40 large T antigen in human primary GPR125-positive spermatogonia from OA patients [44]. Previous studies in our lab and He's lab [33, 45–47] have shown that the SSC line has similar properties to the human primary SSC and that it expresses many SSC markers, including GPR125, GFRA1, PLZF, UCHL1, and THY1, and can be amplified in vitro for a long time. Cell culture medium was DMEM/F12 (Gibco, Grand Island, NY, USA) with 10% fetal bovine serum (FBS; Gibco), and antibiotics were optional. Human SSC lines were passaged every 2-3 days at 34°C and 5% CO_2_.

### 2.4. Protein Extraction, Western Blot Analysis, and Immunoprecipitation Assays

Total proteins from cells and tissues were extracted using RIPA buffer (Thermo Scientific). Briefly, RIPA buffer was added to the samples and well ground, lysed on ice for 15 min. The supernatant was then centrifuged at 12000 g for 15 min. Immunoblots and immunoprecipitation of proteins were performed as previously described [31]. Briefly, approximately 30 mg of total protein was used for SDS-PAGE (Bio-Rad) electrophoresis, and after transfer of the proteins to a polyvinylidene fluoride (PVDF) membrane, the primary antibody was incubated at 4°C for 16 hours, followed by the secondary antibody, and the bands were visualized and analyzed using a chemiluminescence system (Bio-Rad). For immunoprecipitation, the total protein was incubated with primary antibody or rabbit IgG overnight at 4°C. Then, protein G magnetic beads were added to the cell lysates and incubated at 4°C for 2 hours. After washing 3 times with washing buffer, the magnetic beads were precipitated using a magnetic field, resuspended and heated at 95°C for 5 min, and the supernatant was collected for western blot. Details of the antibodies used in the experiments are listed in Supplementary Table 1.

### 2.5. Immunohistochemistry and Immunofluorescence

For immunohistochemistry, testis sections were deparaffinized with xylene, rehydrated with graded ethanol, and then heated in 0.01 M sodium citrate buffer for 18 minutes at 98°C. Then, endogenous peroxidase activities are blocked with 3% H_2_O_2_ (Zsbio, Beijing, China). Next, incubated with 0.25% Triton X-100 for 15 min at room temperature (RT) to permeabilize the tissue, but this is optional. After this, tissues were blocked for one hour at RT with 5% bull serum albumin (BSA). Incubation of tissue sections with primary antibody for at least 16 hours at 4°C is in Supplementary Table 1. After washing three times with PBS, tissue sections were incubated with a secondary antibody for 1 hour at RT. A diaminobenzidine (DAB) kit (Zsbio, Beijing, China) was used to develop the color. For immunofluorescence, Alexa Fluor-conjugated secondary antibodies were incubated at RT for 1 h, followed by 4',6-diamidino-2-phenylindole (DAPI) counterstaining. At the end of the process, image capture and analysis of tissue sections were performed using a Zeiss microscope (Germany, Carl Zeiss).

### 2.6. Source and Transfection of Plasmids

MAGEB2-flag, EGR1-flag, and pCMV3-flag plasmids (Supplementary Figure 1) were provided by Sino Biological (Beijing, China). According to the operation manual, 2.5 *μ*g plasmid was transfected into human SSC lines using the Lipofectamine 3000 transfection reagent (Life Technologies, CA, USA). Transfection efficiency was about 75% by dual transfection with a GFP reporter plasmid. 48 hours after transfection, the expression of proteins and genes was evaluated.

### 2.7. Cell Counting Kit-8 Assay

The proliferation rate of the human SSC line was tested using the CCK8 kit according to the operating manual (Dojindo, Kumamoto, Japan). Briefly, 10% CCK-8 reagent was added to the culture media and incubated for 3 h. Then, the absorbance of the medium at 450 nm was measured using a microplate reader (Thermo Scientific).

### 2.8. The 5-Ethynyl-2'-Deoxyuridine (EdU) Incorporation Assay

According to the operation manual, 50 *μ*M EdU (RiboBio) reagent was added to the cell culture medium and cultured for 12 h. Human SSC lines were washed with DMEM and fixed with 4% PFA. After neutralization with glycine (2 mg/ml), incubate with 0.5% Triton X-100 for 10 min at RT to permeabilize the cells. Apollo was used for color development. Cell nuclei were counterstained with DAPI. Image capture and analysis were performed using a fluorescence microscopy (Zeiss). At least 500 cells were included for statistical analysis.

### 2.9. Flow Cytometry for Apoptosis Detection

Collection of human SSC lines after 48 h of transfection and wash 2 times with ice PBS. After centrifugation, at least 1 million cells were resuspended using Annexin V binding buffer (BD Biosciences, San Jose, CA, USA) according to operating instructions. Subsequently, cells were incubated with 10 *μ*l PI and 5 *μ*l APC-labeled Annexin V reagent for 15 min at RT, protected from light. The stained cells were analyzed using a C6 flow cytometer (BD Biosciences).

### 2.10. TUNEL Assay

After 48 hours of transfection, the cells were assayed for DNA breaks using an in situ cell death detection kit (Roche, Mannheim, Germany). Briefly, human SSC lines were incubated at RT for 15 min using proteinase K (20 mg/ml) and, after washing 3 times with PBS, incubated for 1 hour with dUTP labeling/terminal deoxynucleotidyl transferase (TdT) enzyme buffer, protected from light. DAPI was used to counterstain the nucleus. Image capture and analysis were performed using a fluorescence microscopy (Zeiss). At least 500 cells were counted for statistical analysis.

### 2.11. Molecular Docking Analysis

Molecular docking analysis was performed through an online analysis website (home for researchers, https://www.dockeasy.cn/DockProtein). In brief, we obtained the structural information of the protein molecule through the PDB database or the SWISS-MODEL database. The crystal structure files of the candidate proteins were uploaded to the website to predict the receptor-ligand complex model by rigid docking. The *Z*-score was used as a selection criterion for the prediction results to investigate the mode of interaction between MAGEB2 and EGR1.

### 2.12. Statistical Analysis

Statistical analysis was performed using GraphPad Prism (GraphPad Software, La Jolla, CA, USA, Version 8.0). Descriptive statistics are shown as means ± standard deviation. The *t*-test was chosen after D'Agostino-Pearson omnibus normality tests for each data. *P* < 0.05 indicated significance.

## 3. Results

### 3.1. RNA Expression Profiles of Human Testicular Cells Based on scRNA-seq Analysis

**Figure 1:**
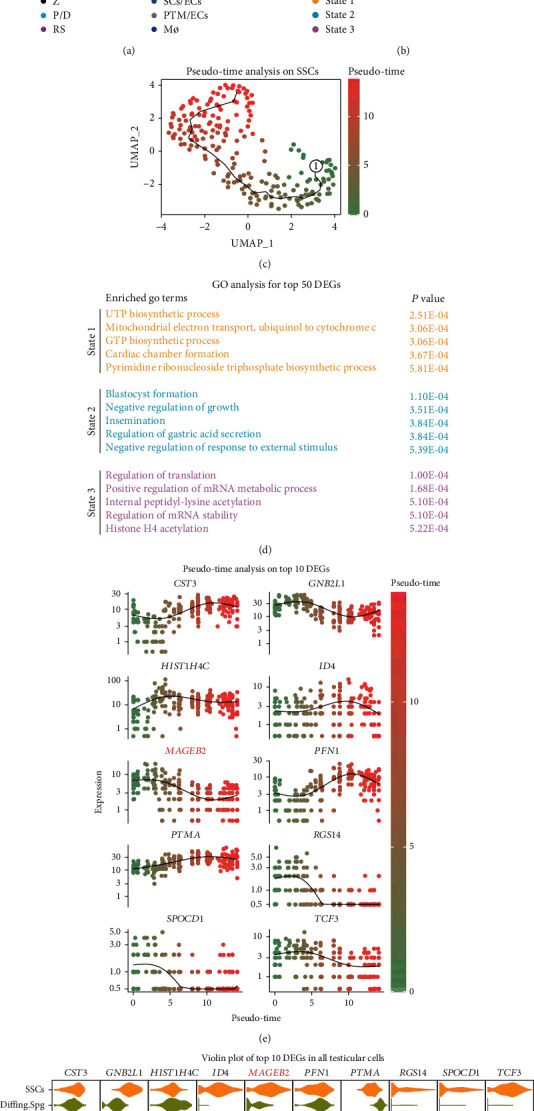
Integrative analysis of human testicular single-cell transcriptional profiles. (a) UMAP clustering and visualization of testicular single-cell RNA expression data. All testicular cells were classified into 12 clusters, of which each point representing an individual cell, and colored according to the type described in the figure legend. (b) Reclustering and visualization of SSC cluster using UMAP. SSCs were categorized into 3 states, states 1, 2, and 3, with each dot representing an individual SSC and colored according to the type described in the figure legend. (c) The developmental trajectory of the SSC population established by pseudotime analysis. Each dot represents an individual SSC, the black curve represents the developmental trajectory of the SSC, and the character 1 represents the developmental starting point. The green color represents the early stage of development, while the red color represents the late stage of development, and all cells are colored according to their developmental status. (d) GO analysis (biological process) of the top 50 DEGs in three different states of SSCs. (e) The expression of the top 10 DEGs in SSC along the developmental trajectory. Each point represents an individual SSC, and the black curve is the mean value of the selected gene at the specified pseudotime. (f) Distribution of SSC top10 DEGs in all testicular cells visualized through violin plot. The plot orientation is set to horizontal. Its horizontal length represents the expression intensity of a specific gene, and the vertical width of a violin plot is proportional to the density of the distribution. UMAP: uniform manifold approximation and projection; GO: Gene Ontology; DEGs: differentially expressed genes.

Adult testicular single-cell RNA sequencing data from GEO datasets (GSE14951236 and GSE11201335) were downloaded and studied to investigate the mechanisms controlling human SSC development. After data filtering and integration using Seurat in R, 4937 testicular cells and 22075 genes were retained, and all cells were classified into 12 clusters. Then, these clusters were identified by assessing the levels of some testicular cell markers, including SSC markers (ID4 and fibroblast growth factor receptor 3 (FGFR3)), differentiating spermatogonia markers (KIT and STRA8), meiosis markers (synaptonemal complex protein 1 (SYCP1), meiotic recombination protein (SPO11), transcription factor ovo-like 2 (OVOL2), and NME/NM23 family member 8 (NME8)), spermatid marker (TNP2 and protamine 2 (PRM2)), and some somatic markers (Supplementary Figure 2). The 12 cell populations are spermatogonial stem cells (SSCs), differentiating spermatogonia (Diffing. Spg), leptotene spermatocytes (L), zygotene spermatocytes (Z), pachytene/diplotene spermatocytes (P/D), round spermatids (RS), elongated spermatocytes (ES), sperm, Leydig cells (LCs), Sertoli cells/endothelial cells (SCs/ECs), peritubular myoid cells/endothelial cells (PTM/ECs), and macrophages (Mø) (Figure 1(a)). To further investigate the developmental mechanisms of SSC, we extracted data from SSC cluster and subdivided them into three states (Figure 1(b)). Then, a pseudotime analysis was performed using the monocle 3 to determine the cell developmental trajectory. According to the levels of piwi-like protein 4 (PIWIL4) and the Nanos homolog 3 (NANOS3), state 3 with a high level of PIWIL4 was considered as the developmental starting point, state 1 with a high level of NANOS3 was believed to be late in development, and state 2 was considered the intermediate transition state (Figure 1(c)). In addition, the top 50 differentially expressed genes (DEGs) of SSCs in states 1, 2, and 3 were analyzed by Gene Ontology (GO), DEGs in state 1 were included in UTP biosynthetic process, DEGs in state 2 were enriched to blastocyst formation and negative regulation of growth, and the DEGs in state 3 were involved in the regulation of translation (Figure 1(d)). The levels of top 10 DEGs in SSCs along with the developmental trajectory were shown using dot plots (Figure 1(e)). The distribution of these genes in all testicular cells was also demonstrated using a violin plot (Figure 1(f)). Among them, MAGEB2 was found to be predominantly expressed in SSC and gradually decreased along the developmental trajectory. These results implied that MAGEB2 may be involved in the fate decision of SSCs.

### 3.2. Validation on the Distribution Pattern of MAGEB2 in Human Testicular Tissue

**Figure 2:**
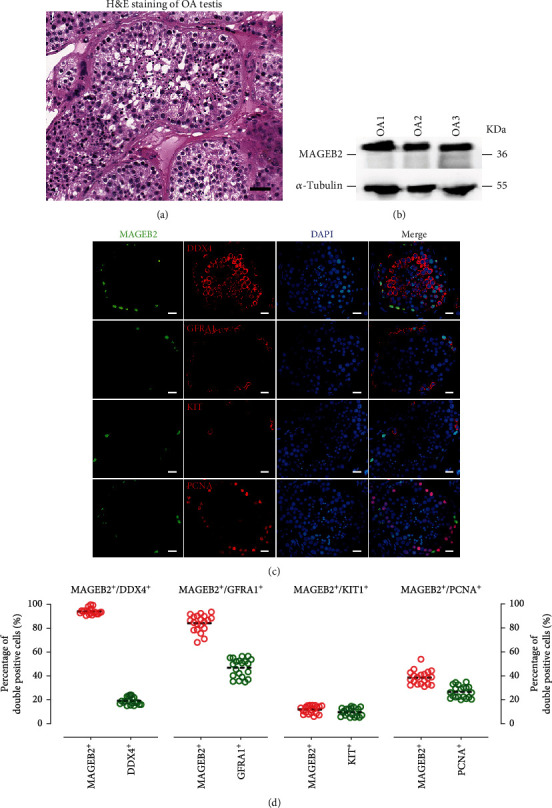
Expression of MAGEB2 in OA patients with normal spermatogenesis. (a) Representative images of HE staining for testicular tissue from patients with OA. Scale bar, 200 *μ*m. (b) Western blot assay to verify the expression of MAGEB2 in three OA patients. (c) Two-color immunofluorescence staining of MAGEB2-expressing cells. MAGEB2 signals are shown in green, signals of different cellular markers including DDX4, GFRA1, KIT, and PCNA are shown in red, and DAPI-labeled nuclei are shown in blue. Scale bar, 20 *μ*m. (d) Dot plots demonstrated the coexpression abundance of MAGEB2 with DDX4, GFRA1, KIT, and PCNA. The *y*-axis is the proportion of double positive cells, each circle represents the result of counting a circular cross section of a seminiferous tubule, and at least 20 seminiferous tubules were counted.

To further validate our findings in the scRNA sequence, we investigated MAGEB2 expression in human testicular tissue using immunofluorescence and western blot. We collected testicular tissue from 3 patients with OA, and the results of the HE stain showed normal spermatogenesis (Figure 2(a)). Then, the overall expression level of MAGEB2 in testicular tissues was detected using western blot, and the results of band visualization indicated that MAGEB2 was detectable in testicular tissues at a high level (Figure 2(b)). We further detected the distribution of MAGEB2 in different testicular cells by two-color immunofluorescence (Figure 2(c)). By costaining with different cellular markers, we found that 94.0% ± 19.2% MAGEB2-positive cells colocalized with DDX4 (germ cell marker), 84.3% ± 7.0% MAGEB2-positive cells expressed GFRA1 (SSC marker), and only 11.8% ± 3.2% MAGEB2-positive cells colocalized with KIT (differentiated spermatogonia marker). Furthermore, only 38.5% ± 5.7% of MAGEB2-positive cells weakly expressed PCNA (cell proliferation marker), implying that MAGEB2 can negatively regulate proliferation (Figure 2(d)). These results are consistent with scRNA-seq analysis and suggested that MAGEB2 was predominantly expressed in human SSC and may negatively regulate SSC proliferation.

### 3.3. The Effects of MAGEB2 on the Proliferation of Human SSC Lines

**Figure 3:**
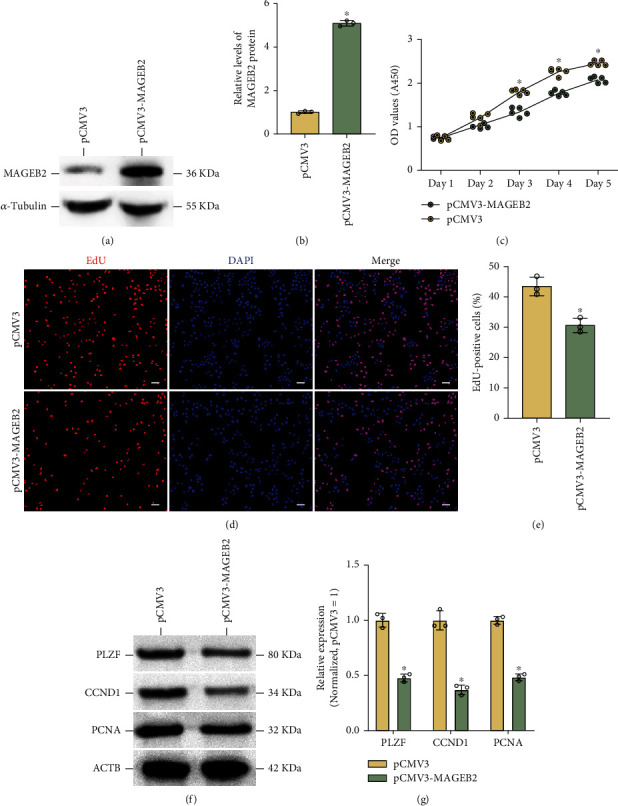
MAGEB2 overexpression inhibited the proliferation of SSC lines. (a) Western blot assay to verify the overexpression of MAGEB2. (b) The bar graph showed the relative levels of MAGEB2 after overexpression in the SSC lines. (c) The CCK8 assay detected cell proliferation on days 1-5 of MAGEB2 overexpression, with a significant decrease in cell proliferation from day 3. (d) EdU assay examined cell DNA synthesis after MAGEB2 overexpression. (e) The bar graph demonstrates the relative changes in cellular DNA synthesis following MAGEB2 overexpression. Scale bar, 50 *μ*m. (f) Western blot experiments showed expression changes of proteins related to SSC proliferation, such as PLZF, CCND1, and PCNA. (g) The bar graph showed the relative levels of PLZF, CCND1, and PCNA after MAGEB2 overexpression in the SSC lines. OD: optical density; A450: absorbance at 450 nm. ^∗^ represents *P* < 0.05.

To further investigate the effect of MAGEB2 on human SSC proliferation, the SSC line, which has similar characteristics to human primary cells, was applied to study the effects of MAGEB2 on cell proliferation and apoptosis. Taking into account the low expression of MAGEB2 in the cell lines, we overexpressed MAGEB2 in the SSC line using plasmids (pCMV3-MAGEB2), and the results of western blot showed that the level of MAGEB2 in the SSC line was significantly elevated after overexpression (Figures 3(a)and 3(b)). Then, we examined cell proliferation from day 1 to day 5 after transfection using CCK8 assay, and the proliferation of cells after MAGEB2 overexpression was significantly decreased from day 3 to day 5 compared to the control group (Figure 3(c)). Likewise, we examined DNA synthesis using the EdU assay 48 h after transfection, and the results showed that MAGEB2 overexpression significantly inhibited DNA synthesis compared to the control group (30.6% ± 2.4% vs. 43.4% ± 3.0%, *P* < 0.05) (Figures 3(d) and 3(e)). In addition, we examined several proteins associated with SSC proliferation and self-renewal through a western blot assay, including PLZF, CCND1, and PCNA, and the results showed that the upregulation of MAGEB2 suppressed the expression of these proteins (Figures 3(f) and 3(g)). These data indicated that MAGEB2 overexpression weakened the proliferation of SSC lines.

### 3.4. The Impacts of MAGEB2 Upregulation on SSC Line Apoptosis

**Figure 4:**
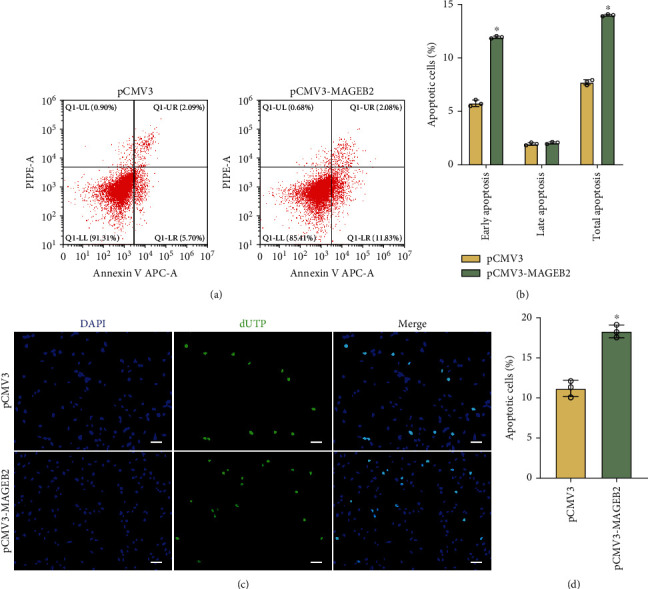
MAGEB2 overexpression caused apoptosis in the SSC lines. (a) Flow cytometry combined with Annexin V staining to detect apoptosis in SSC lines. (b) The bar plot shows the results of apoptosis detected by flow cytometry. (c) TUNEL staining detects DNA breaks in the SSC lines. Green represents the presence of breaks in the cellular DNA, and blue represents the DAPI-labeled nucleus. Scale bar, 50 *μ*m. (d) The bar graph shows the results of the TUNEL staining in (c). ^∗^ represents *P* < 0.05.

After 48 h of plasmid transfection, we found that cell fragmentation was significantly higher in the MAGEB2 group than that in the control group, which implies an increase in apoptosis. To examine whether MAGEB2 upregulation promotes apoptosis in SSC lines, flow cytometry was applied. The results of Annexin V staining showed that MAGEB2 overexpression resulted in a significant upregulation of apoptosis levels (early apoptosis: 5.8% ± 0.3% vs.11.9% ± 0.1%, *P* < 0.05; late apoptosis: 1.9% ± 0.1% vs. 2.1% ± 0.1%, *P* < 0.05) (Figures 4(a) and 4(b)). Similarly, the results of TUNEL staining were consistent with flow cytometry, and overexpression of MAGEB2 in the SSC lines caused an increase in cellular DNA breaks (11.2% ± 1.0% vs 18.3% ± 0.8%, *P* < 0.05) (Figures 4(c) and 4(d)). These data showed that upregulation of MAGEB2 induced apoptosis in the SSC lines.

### 3.5. Prediction and Validation of MAGEB2-Interacting Proteins

**Figure 5:**
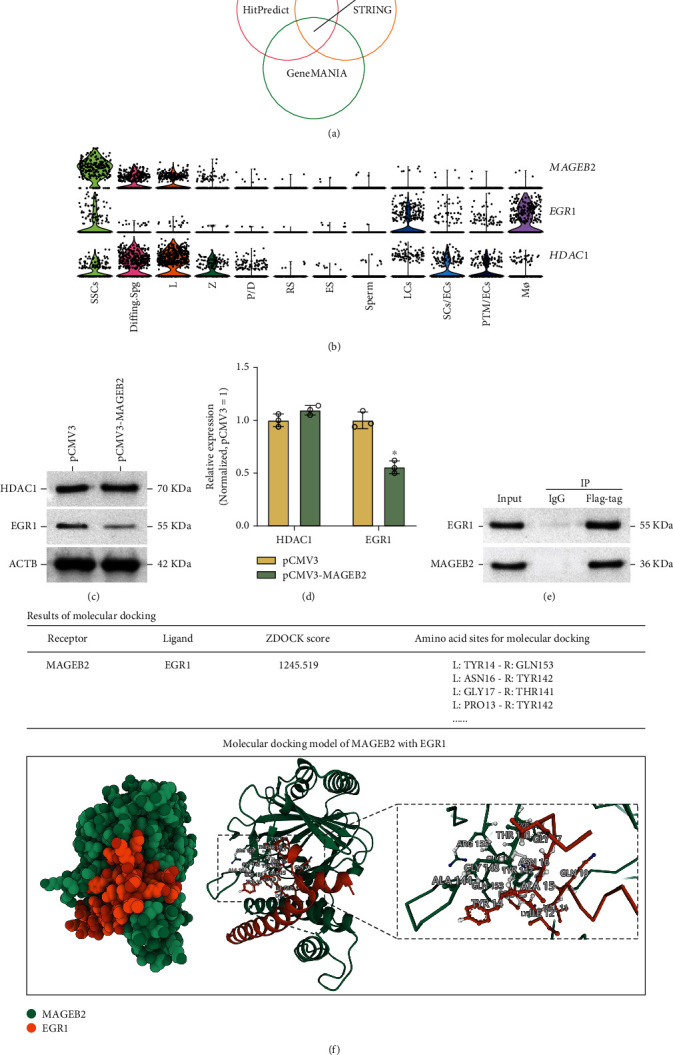
Validation of MAGEB2 candidate target proteins in SSC lines. (a) Prediction of MAGEB2 candidate target proteins using HitPredict, STRING, and GeneMANIA databases. Venn diagram showing the intersection of the predictions from the 3 databases. (b) The violin plot demonstrates the distribution of *MAGEB2* and the candidate target proteins *EGR1* and *HDAC1* in testicular cells at the mRNA level. Each point in the plot represents a cell sample, the *y*-axis represents the expression intensity of the mRNA, and the *x*-axis denotes the probability density of the gene in the cell population. (c) Western blot analysis of EGR1 and HDAC1 expression at the protein level after overexpression of MAGEB2. (d) The bar graph shows the expression levels of EGR1 and HDAC1 after MGEB2 upregulation. (e) Protein immunoprecipitation assay verified the interaction of MAGEB2 with EGR1. (f) Results and models of molecular docking. Rigid protein–protein docking (ZDOCK) was performed between MAGEB2 and EGR1 to study the relationships. The amino acid sites of protein molecule interactions are shown in the table and the ball-and-stick diagram, such as TYR14-GLN153 and ASN16-TYR142. TYR14 represents tyrosine at position 14 of the peptide chain, and the others are similar. ^∗^ indicates *P* < 0.05. L: ligand; R: receptor; TYR: tyrosine; GLN: glutamine; ASN: asparagine; GLY: glycine; THR: threonine; PRO: proline.

MAGEB2 has been reported to be an ubiquitin enhancer that promotes the degradation of certain proteins by ubiquitination [48]. Taking this into account, we predicted MAGEB2 target proteins using the GeneMANIA, STRING, and HitPredict databases. After excluding the known ubiquitin ligases, MAGEB2 was predicted to bind to EGR1 and HDAC1 (Figure 5(a)). Then, we examined the distribution patterns of *EGR1* and *HDAC1* in the scRNA-seq results, and the violin graph showed that both *EGR1* and *HDAC1* were coexpressed with *MAGEB2*, but EGR1 expression was more restricted, and it was coexpressed with *MAGEB2* in the SSC lines (Figure 5(b)). Subsequently, we examined the interaction of MAGEB2 with EGR1 and HDAC1 at the protein level. In SSC lines, MAGEB2 overexpression led to a decrease in EGR1 but not HDAC1 (Figures 5(c) and 5(d)). It implied that MAGEB2 might not affect HDAC1 degradation in SSC lines. The results indicated that MAGEB2 could bind to EGR1 (Figure 5(e)). Furthermore, the results of molecular docking also showed that MAGEB2 was rigidly docked with EGR1 with a *Z*-score of 1245.519, and EGR1 formed links with MAGEB2 at amino acid sites such as TYR14-GLN153 and ASN16-TYR142 (Figure 5(f)). These results suggested that MAGEB2 interacts with EGR1 in the SSC lines.

### 3.6. EGR1 Is Responsible for the Decreased Proliferation of SSC Lines by MAGEB2 Overexpression

**Figure 6:**
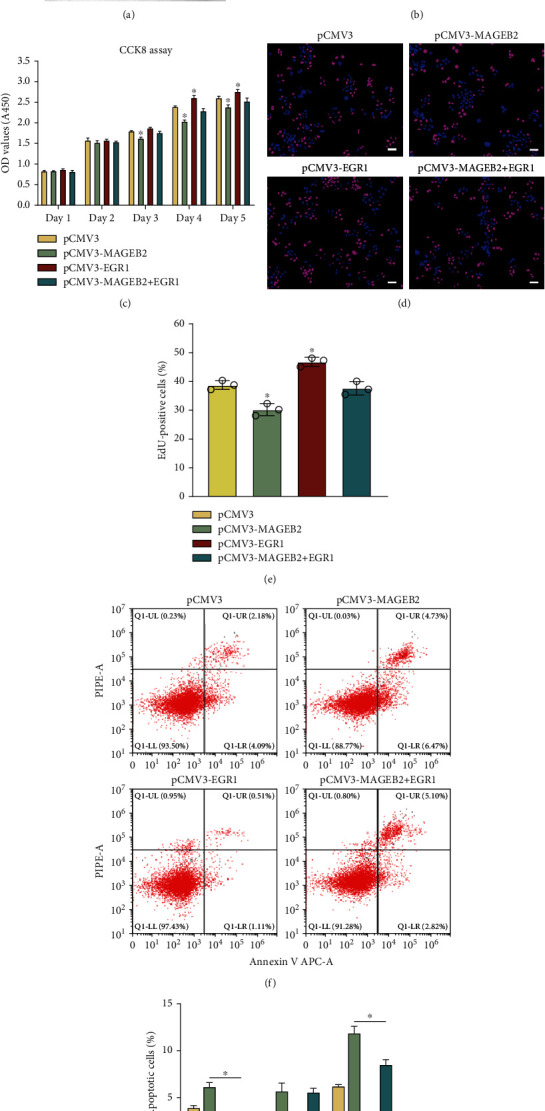
Rescue effects of EGR1 on the proliferation of MAGEB2-overexpressed SSC lines. (a) Western blot detects the protein levels of PLZF, EGR1, MAGEB2, and PCNA after overexpression of MAGEB2, EGR1, or both. (b) The bar graph displays the levels of PLZF, EGR1, MAGEB2, and PCNA after transfection. (c) CCK8 assay detects the proliferation of SSC lines transfected with expression plasmids of MAGEB2, EGR1, or both. (d) EdU assay examines cellular DNA synthesis after transfection. Scale bar, 50 *μ*m. (e) The bar graph shows the percentage of EdU positive cells. (f) Annexin V staining combined with FACS detects apoptosis of SSC lines. (g) The bar plot demonstrates the percentage of early, late, and total apoptotic cells. ^∗^ indicates *P* < 0.05.

To verify whether EGR1 plays a role in MAGEB2-mediated inhibition of SSC line proliferation, we transfected SSC lines with expression plasmids of MAGEB2, EGR1, or both to observe the proliferation and apoptotic changes. The expressions of MAGEB2 and EGR1 were measured using western blot at 48 h posttransfection to verify the efficiency of transfection, and we also found that the levels of PLZF and PCNA were reversed after reexpression of EGR1, suggesting that cell proliferation was restored (Figures 6(a) and 6(b)). After this, we examined cell proliferation using CCK8 after transfection from day 1 to day 5. The results showed that upregulation of EGR1 promoted proliferation of SSC lines and partially rescued the MAGEB2-mediated proliferation inhibition (Figure 6(c)). The EdU assay yielded similar results, with DNA synthesis in the cells partially restored after EGR1 was overexpressed (Figures 6(d) and 6(e)). Furthermore, we also detected changes in apoptosis by FACS, and the results demonstrated that reexpression of EGR1 partially rescued the apoptosis of SSC lines caused by MAGEB2 overexpression (Figures 6(f) and 6(g)). These data indicated that EGR1 plays a role in MAGEB2-mediated cell proliferation and apoptosis, suggesting that EGR1 is a downstream target of MAGEB2.

### 3.7. MAGEB2 Downregulation May Be Correlated with Spermatogenic Failure in NOA Patients

**Figure 7:**
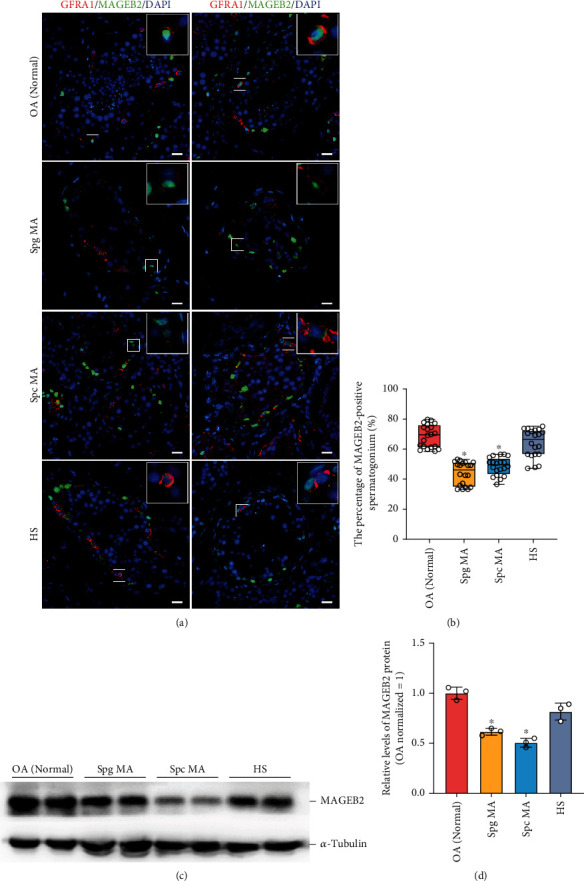
Distribution of MAGEB2 in patients with OA and NOA. (a) Distribution of MAGEB2 in SSC of different testicular tissues detected by dual-color immunofluorescence: the green color is MAGEB2 signal, the red color is GFRA1-labeled SSC signal, and the blue color is DAPI-labeled nucleus. Representative images are shown at the top right with partially enlarged details. Scale bars = 50 *μ*m. (b) Box plots showing the proportion of MAGEB2-positive SSCs in different samples. Each circle in the box plot represents one result, and at least 20 cross-sections of the seminiferous tubule were counted. (c) Western blot detected the expression of MAGEB2 in different testicular samples. (d) The bar graph demonstrated the relative levels of MAGEB2 in different testicular tissues. OA: obstructive azoospermia with normal spermatogenesis. Spg MA: spermatogonia maturation arrest. Spc MA: spermatocyte maturation arrest. HS: hypospermatogenesis. ^∗^ indicates *P* < 0.05.

NOA is one of the most severe and hard-to-treat forms of male infertility. To determine whether dysregulation of spermatogenesis is related to MAGEB2 levels, we collected testicular samples from eight patients who underwent mTESE and assessed their spermatogenesis status by HE staining; these samples were categorized as OA, spermatogonia maturation arrest (Spg MA), spermatocyte maturation arrest (Spc MA), and hypospermatogenesis (HS) (Supplementary Figure 3). Considering that normal adult testes are difficult to obtain, we used two OA samples with normal spermatogenesis as normal controls. Then, a two-color immunofluorescence was used to analyze the distribution of MAGEB2 in the SSC of different testicular samples, and the results showed that the proportion of MAGEB2-positive SSC decreased in the sample with abnormal spermatogenesis, especially in patients with Spg MA and Spc MA (Figures 7(a) and 7(b)). Consistent with the result of immunofluorescence, the results of western blot showed that the total level of MAGEB2 was significantly downregulated in the samples of Spg MA and Spc MA (Figures 7(c) and 7(d)). These results implied that aberrant levels of MAGEB2 may be correlated with dysregulated spermatogenesis, particularly the occurrence of Spg MA and Spc MA.

## 4. Discussion

SSCs are the basis for male spermatogenesis. In mice, Brinster and Zimmermann first achieved the restoration of spermatogenesis in infertile mice by transplantation of undifferentiated spermatogonia [49]. In 2000, Meng et al. identified a key role for GDNF in SSC self-renewal [8]. Later, Kanatsu-shinohara et al. established a stable in vitro culture system for mouse SSCs [9]. These critical advances have facilitated SSC-related research. However, the results of mouse SSC studies cannot be fully applied to humans due to species differences. Human SSCs were difficult to culture in vitro for long due to its weak proliferative capacity. Using human testes samples as a research sample to directly investigate the development mechanism of SSC is a crucial way to establish an in vitro culture system for human SSCs.

The advent of scRNA-seq allows us to observe transcriptome changes in human SSCs more clearly at the level of individual cells, which helps to understand the developmental process of SSCs. We obtained a transcriptional profile of testicular cells by integrating and analyzing two published human testicular scRNA-seq datasets [40, 41] and further identified many genes that may be involved in regulating SSCs. MAGEB2 is predominantly expressed in human SSCs and gradually decreases along the developmental trajectory, suggesting that it may play a role in SSC self-renewal. This is consistent with the immunohistochemical findings that MAGEB2 is expressed in the nucleolus and nucleoplasm of SSCs but is rarely present in differentiated spermatogonia.

MAGEB2 is a member of the melanoma antigen gene family member [48]. It is reported that MAGEB2 binding to androgen receptor promotes prostate cancer proliferation through upregulation of PSA and NX3.1 [50]. MAGEB2 binds to histone deacetylase 1 (HDAC1) and activates the transcription factor E2F (E2F) that stimulates the proliferation of colon cancer cells [51]. MAGEB2 was reported to be related to the progression of squamous cell carcinoma [52] and breast cancer [53]. Our results showed that upregulation of MAGEB2 inhibited the proliferation of human SSC lines and induced their apoptosis. This differs from the roles reported in tumors. Therefore, we hypothesized that MAGEB2 could have different target proteins in SSCs, which resulted in MAGEB2 inhibiting rather than promoting SSC proliferation. Using protein interaction prediction, molecular docking, and immunoprecipitation analysis, we found that EGR1 interacts with MAGEB2, and this was supported by the results of rescue experiments. However, we need to determine whether MAGEB2 directly interacts with EGR1. MAGE family proteins bind to the RING domain of E3 ubiquitin ligases, stabilizing their structure and influencing the degradation process of downstream proteins [48]. For example, the melanoma-associated antigen C2 (MAGEC2) functions as a tumorigenic gene by interacting with the TIRM28 to promote cellular tumor antigen p53 degradation [48]. Therefore, we speculated that MAGEB2 might interact with EGR1 through E3 protein ligase to promote EGR1 degradation. In addition, we cannot exclude the possibility that MAGEB2 can regulate the proliferation of the SSC lines through other unknown molecules. More experimental results, such as immunoprecipitation and protein mass spectrometry, are needed to answer these questions. Furthermore, we found that the upregulation of MAGEB2 led to significant downregulation of PLZF, CCND1, and PCNA. Whether they are directly regulated by ubiquitination involving MAGEB2 needs to be further verified.

In our study, we examine the distribution and total amount of MAGEB2 in different testicular tissues. Tissues from patients with Spg MA and Spc MA exhibited a significant downregulation of MAGEB2, suggesting that MAGEB2 may be associated with dysregulated spermatogenesis. EGR1 has been reported to be involved in the proliferation of various stem cells and tumor cells, including hematopoietic stem cells [54, 55], neural stem cells [56], gastric cancer cells [57], and prostate cancer cells [58]. As a target protein of MAGEB2 in human SSC lines, we do not know whether EGR1 is also aberrantly distributed in the testes of NOA patients, and we will examine it in future experiments. Notably, it has been recently reported that the ablation of MAGEB2 in the testis does not affect the fertility of male mice in a normal physiological state, but the tolerance to thermal stimulation was impaired and thus led to abnormal spermatogenesis [59]. This study provided evidence that MAGEB2 deficiency affects male spermatogenesis. However, its role in human spermatogenesis still needs to be confirmed. In the future, we will screen for potentially MAGEB2 mutations in NOA patients and verify their function using point mutation models in cells and animals.

## 5. Conclusion

**Figure 8:**
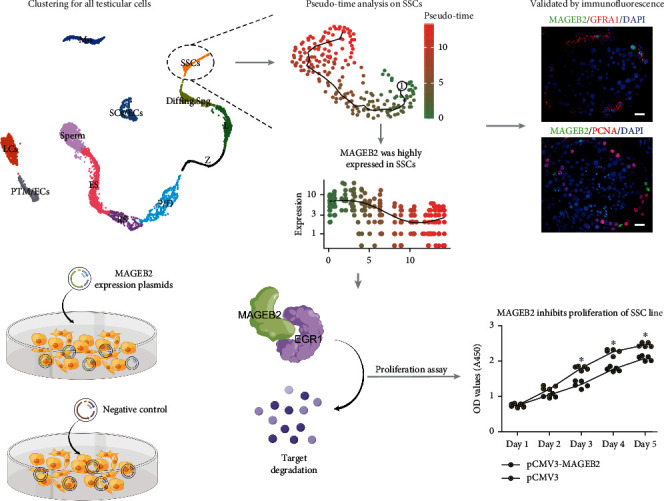
The graphical summary of this study. Our study identified the dominant expression of MAGEB2 in human SSC by scRNA RNA analysis and immunohistochemistry and used cellular and molecular experiments to reveal that MAGEB2 mediates the degradation of EGR1 to regulate the proliferation and apoptosis of the human SSC lines.

In summary, our data indicated that MAGEB2 was predominantly expressed in human SSCs. MAGEB2 affects the proliferation and apoptosis of human SSC lines by regulating the degradation of EGR1 (Figure 8). Meanwhile, MAGEB2 was found to be significantly downregulated in testicular tissue from Spg MA and Spc MA patients, implying that it is associated with abnormal spermatogenesis. Our results contribute to the understanding of the regulation of proliferation and self-renewal of human SSCs and may provide new ideas for the treatment of male infertility.

## Data Availability

The data generated by this study can be obtained from the corresponding author upon reasonable request.

## Supplementary Materials

Supplementary Materials Supplementary Figure 1: genetic maps of MAGEB2 and EGR1 expression plasmid inserts. Supplementary Figure 2: expression patterns of selected markers are projected on the UMAP plot. (a) UMAP plot showing expression patterns of selected markers. As shown in the figure key, blue indicates high expression, while gray indicates low or no expression. Spermatogonial stem cells are identified by ID4, and FGFR3, KIT, and STRA8 are differentiating spermatogonia markers; SYCP1, SPO11, OVOL2, NME8, and ZPBP are meiosis markers, TNP2 and PRM2 are spermatids and sperm markers, endothelial cells are identified by CD34, WT1 is a marker for Sertoli cells, IGF1 is a marker for Leydig cells, peritubular myoid cells are identified by MYH11, and macrophages are identified by CD68. (b) UMAP and clustering analysis of all human testicular cells. Supplementary Figure 3: H&E staining for 8 testicular tissues with different spermatogenic statuses. All samples were classified as OA, Spg MA, Spc MA, and HS according to the Johnsen score. Scale bars, 50 *μ*m. Supplementary Table 1: antibodies applied in western blots, immunofluorescence, and immunoprecipitation.
