# Supplementary material for: MAGEB2-Mediated Degradation of EGR1 Regulates the Proliferation and Apoptosis of Human Spermatogonial Stem Cell Lines
Source: Stem Cells Int. 2023 Jun 2;2023:3610466. doi: 10.1155/2023/3610466 (PMC10256451; doi:10.1155/2023/3610466)
Supplement: Supplementary Materials — Supplementary Figure 1: genetic maps of MAGEB2 and EGR1 expression plasmid inserts. Supplementary Figure 2: expression patterns of selected markers are projected on the UMAP plot. (a) UMAP plot showing expression patterns of selected markers. As shown in the figure key, blue indicates high expression, while gray indicates low or no expression. Spermatogonial stem cells are identified by ID4, and FGFR3, KIT, and STRA8 are differentiating spermatogonia markers; SYCP1, SPO11, OVOL2, NME8, and ZPBP are meiosis markers, TNP2 and PRM2 are spermatids and sperm markers, endothelial cells are identified by CD34, WT1 is a marker for Sertoli cells, IGF1 is a marker for Leydig cells, peritubular myoid cells are identified by MYH11, and macrophages are identified by CD68. (b) UMAP and clustering analysis of all human testicular cells. Supplementary Figure 3: H&E staining for 8 testicular tissues with different spermatogenic statuses. All samples were classified as OA, Spg MA, Spc MA, and HS according to the Johnsen score. Scale bars, 50 μm. Supplementary Table 1: antibodies applied in western blots, immunofluorescence, and immunoprecipitation. [file 3610466.f1.pdf]

Supplementary information

MAGEB2 inhibits the proliferation of human spermatogonial stem cells

Supplemental Data:
3 Figures
1 Table

Supplemental Figures

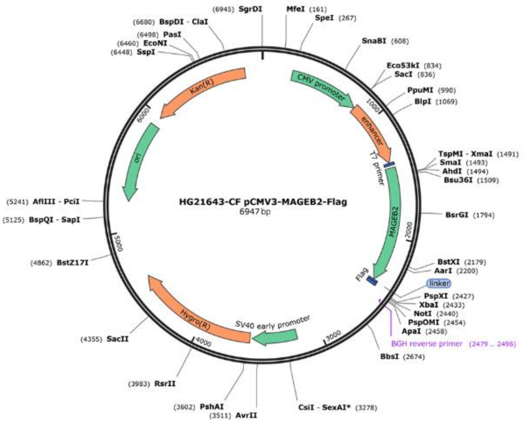

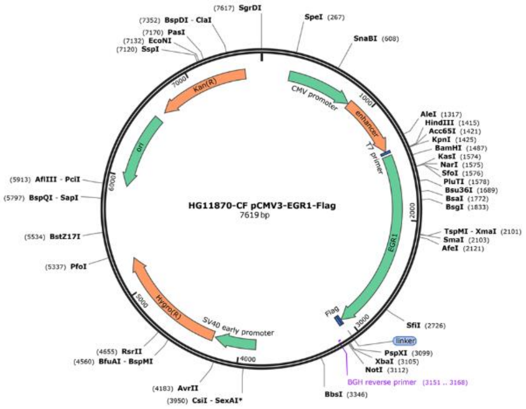

Supplementary **Figure 1: Genetic maps of MAGEB2 and EGR1 expression plasmid inserts.**

Supplementary Figure 2

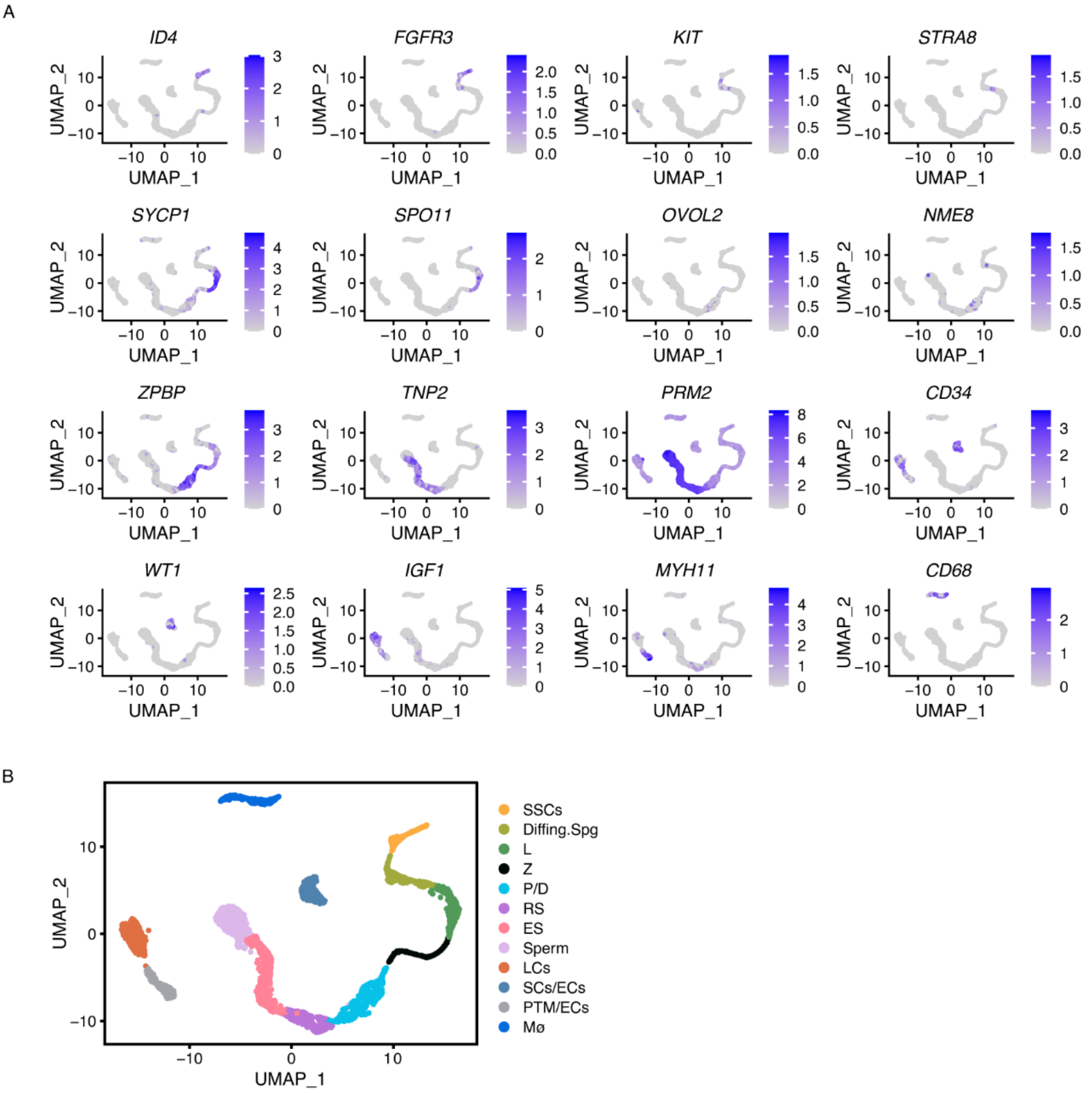

Supplementary Figure 2. Expression patterns of selected markers are projected on the UMAP plot. (A) UMAP plot showing expression patterns of selected markers. As shown on the figure key, blue indicates high expression, while gray indicates low or no expression. Spermatogonial stem cells are identified by *ID4* and *FGFR3*, *KIT* and *STRA8* are differentiating spermatogonia markers, *SYCP1*, *SPO11*, *OVOL2*, *NME8* and *ZPBP* are meiosis markers, *TNP2* and *PRM2* are spermatids and sperm markers, Endothelial cells are identified by *CD34*, WT1 is a marker for Sertoli cells, *IGF1* is a marker for Leydig cells, Peritubular myoid cells are identified by *MYH11*, and macrophages are identified by *CD68*. (B) UMAP and clustering analysis of all human testicular cells.

Supplementary Figure 3

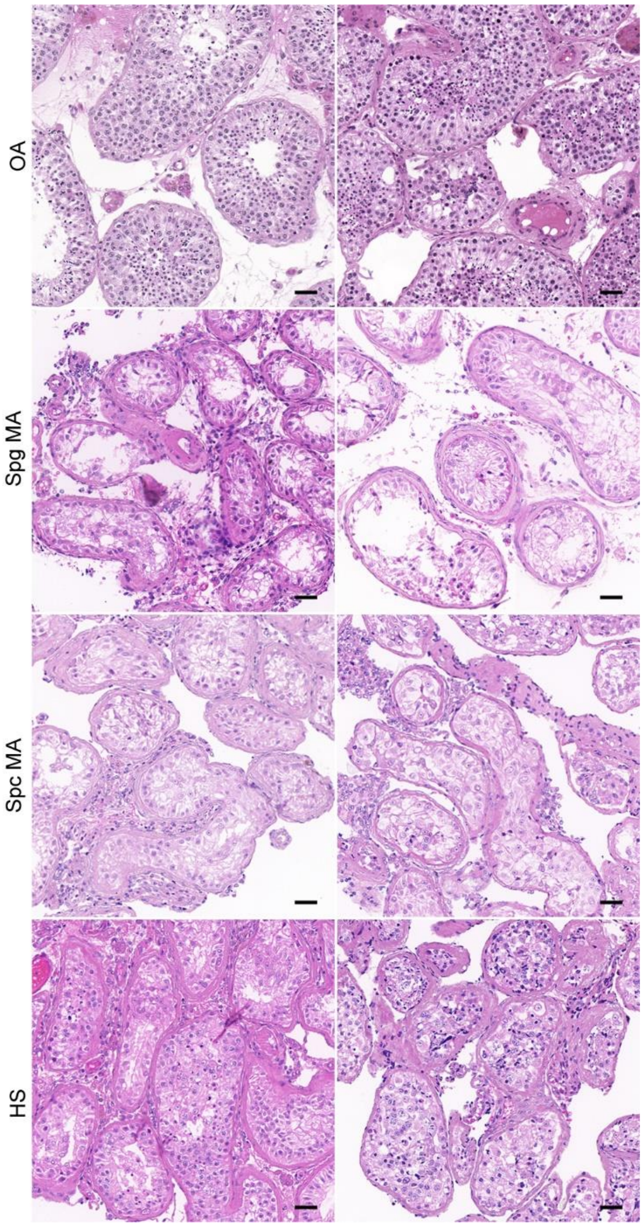

Supplementary Figure 3: H&E staining for 8 testicular tissues with different spermatogenic statuses. All samples were classified as OA, Spg MA, Spc MA, and HS according to the Johnsen score. Scale bars, 50μm.

Supplementary Table 1. Antibodies applied in Western blots, immunofluorescence and immunoprecipitation

Antibodies	Source	Dilution	Incubation
Western blot			
MAGEB2	Sigma# HPA074544	1:500	12h at 4°C
PLZF	SantaCruz cat#sc-28319	1:1000	12h at 4°C
PCNA	Abcam cat#ab29	1:500	12h at 4°C
CCND1	Sigma#SAB1405510	1:800	12h at 4°C
ACTB	Cwbio cat#CW0096	1:2000	12h at 4°C
α-Tubulin	Sigma cat#T9026	1:1000	12h at 4°C
EGR1	Sinobiological cat#100899	1:800	12h at 4°C
Immunoprecipitation			
FLAG	Abcam cat#ab205606	1:25	16h at 4°C
Immunofluorescence			
MAGEB2	Sigma# HPA074544	1:50	16h at 4°C
DDX4	Abcam cat#ab27591	1:50	16h at 4°C
GFRα1	R&Dcat#AF560	1:25	16h at 4°C
PCNA	Abcam cat#ab29	1:50	12-16h at 4°C
KIT	R&D cat#AF332	1:25	16h at 4°C
Secondary Antibody			
Donkey anti-Rabbit IgG, Alexa Fluor 488	Thermo Fisher Scientific cat#A21206	1:1000	1h at 25°C
Donkey anti-Mouse IgG, Alexa Fluor 594	Thermo Fisher Scientific cat#A21203	1:1000	1h at 25°C
Donkey anti-Goat IgG, Alexa Fluor 594	Thermo Fisher Scientific cat#A11058	1:1000	1h at 25°C
